# Identifying Functions of Proteins in Mice With Functional Embedding Features

**DOI:** 10.3389/fgene.2022.909040

**Published:** 2022-05-16

**Authors:** Hao Li, ShiQi Zhang, Lei Chen, Xiaoyong Pan, ZhanDong Li, Tao Huang, Yu-Dong Cai

**Affiliations:** ^1^ College of Biological and Food Engineering, Jilin Engineering Normal University, Changchun, China; ^2^ Department of Biostatistics, University of Copenhagen, Copenhagen, Denmark; ^3^ College of Information Engineering, Shanghai Maritime University, Shanghai, China; ^4^ Institute of Image Processing and Pattern Recognition, Shanghai Jiao Tong University, and Key Laboratory of System Control and Information Processing, Ministry of Education of China, Shanghai, China; ^5^ Bio-Med Big Data Center, CAS Key Laboratory of Computational Biology, Shanghai Institute of Nutrition and Health, University of Chinese Academy of Sciences, Chinese Academy of Sciences, Shanghai, China; ^6^ CAS Key Laboratory of Tissue Microenvironment and Tumor, Shanghai Institute of Nutrition and Health, University of Chinese Academy of Sciences, Chinese Academy of Sciences, Shanghai, China; ^7^ School of Life Sciences, Shanghai University, Shanghai, China

**Keywords:** mouse protein, multi-label classification, embedding features, rakel, feature selection

## Abstract

In current biology, exploring the biological functions of proteins is important. Given the large number of proteins in some organisms, exploring their functions one by one through traditional experiments is impossible. Therefore, developing quick and reliable methods for identifying protein functions is necessary. Considerable accumulation of protein knowledge and recent developments on computer science provide an alternative way to complete this task, that is, designing computational methods. Several efforts have been made in this field. Most previous methods have adopted the protein sequence features or directly used the linkage from a protein–protein interaction (PPI) network. In this study, we proposed some novel multi-label classifiers, which adopted new embedding features to represent proteins. These features were derived from functional domains and a PPI network via word embedding and network embedding, respectively. The minimum redundancy maximum relevance method was used to assess the features, generating a feature list. Incremental feature selection, incorporating RAndom k-labELsets to construct multi-label classifiers, used such list to construct two optimum classifiers, corresponding to two key measurements: accuracy and exact match. These two classifiers had good performance, and they were superior to classifiers that used features extracted by traditional methods.

## 1 Introduction

Protein is a major component associated with the maintenance of normal physical functions in cells (Milo, 2013). As the essential regulator and effector for almost all living creatures with cellular structures, proteins participate in physical biological processes in two major approaches (Aebersold and Mann, 2016). First, proteins contribute to the regulation of essential biological functions. According to recent publications, proteins are associated with various biological processes, including cell proliferation (Üretmen Kagıalı et al., 2017), enzyme-mediated metabolic processes (Davidi and Milo, 2017), DNA replication (Mughal et al., 2019), cell signaling via ligand binding (Hotamisligil and Davis, 2016), and responses to internal or external stimulations (Chivasa and Slabas, 2012), all of which are quite complex and essential functions for living creatures. In addition, proteins can construct basic cellular structures (Aebersold and Mann, 2016), maintain the stability of cellular microenvironment, and participate in the formation of complex macrostructures of living creatures, such as hairs and nails. Considering the significance of proteins for living creatures, their biological functions and related detailed underlying mechanisms have been widely studied as an irreplaceable field in current biological studies.

Different kinds of proteins in humans are generated by 19823 predicted or confirmed protein-coding genes (Beck et al., 2011; Milo, 2013). For mouse, as a widely used experimental model, several proteins are translated from approximately 12300 specific protein-coding genes and their isoforms (Church et al., 2009). Therefore, considering the large number of proteins in humans and mice, exploring protein functions by analyzing all candidate proteins one by one through traditional experiments is impossible. For the systematic study of protein functions, computational methods and databases are introduced. Early in 2004, Ruepp et al. have already presented an effective and simplified annotation scheme for systematic classification of proteins (Ruepp et al., 2004). Using such annotation tools, proteins can be clustered into 24 functional categories. The final summary of these 24 categories is generated by balancing manual operative convenience, categorial specificity, and adaptability for further downstream analyses. Therefore, annotating proteins with these 24 categories may be an efficient and convenient way for the exploration of initial protein function.

However, in the presence of clusters and related annotated proteins, computational methods for classification may also be necessary for further systematic protein function explorations. In 2011, Hu et al. proposed two computational methods, namely, network-based and hybrid-property methods, to identify the functions of mouse proteins among the aforementioned 24 categories (Hu et al., 2011). The final method integrated these two methods in a way that the network-based method was initially applied to make prediction; if this method cannot provide predicted results, then the hybrid-property method would make further prediction. In addition, Huang et al. provided three computational methods for the prediction of mouse protein functions based on the 24 candidate categories (Huang et al., 2016). Considering the biochemical properties of proteins and specific functioning approaches for most proteins via protein–protein interactions (PPI), three methods were presented for functional annotation/prediction: 1) sequence similarity-based prediction, 2) weighted PPI-based prediction, and 3) sequence recoding-based prediction using PseAAC (Shen and Chou, 2008). The two above-mentioned studies all used mouse proteins and their functional categories reported in the Mouse Functional Genome Database (MfunGD, http://mips.gsf.de/genre/proj/mfungd/) (Ruepp et al., 2006). However, the above-mentioned methods were not absolute multi-label classifiers as they can only provide the category sequence. Moreover, determining predicted categories for a query protein remains a problem. This study continued doing some work in this field. Furthermore, Zhang et al. developed I-TASSER/COFACTOR method for neXtProt project to predict GO functions of proteins based on their structures and interactions (Zhang et al., 2018; Zhang et al., 2019). NetGO (https://issubmission.sjtu.edu.cn/ng2/) predicted protein functions by integrating massive sequence, text, domain/family and network information with Naïve GO term frequency, BLAST-KNN, LR-3mer, LR-InterPro, LR-ProFET, Net-KNN, LR-text and Seq-RNN (You et al., 2019; Yao et al., 2021).

This study also adopted mouse proteins and their function annotations reported in MfunGD. For each protein, we extracted features from two aspects. On the one hand, embedding features derived from functional domains of proteins were extracted, which can indicate the essential properties of proteins. The functional domains were retrieved from the InterPro database (Blum et al., 2021), and features were obtained by a natural language processing method, Word2vec (Mikolov et al., 2013; Church, 2017). On the other hand, other embedding features were obtained from a PPI network, which contained the linkage information to other proteins. We used the PPI network reported in STRING (Szklarczyk et al., 2015), and Node2vec (Grover and Leskovec, 2016) was applied to such network to obtain embedding features. Embedding features were collected to represent all mouse proteins. Afterward, a feature selection procedure, including the minimum redundancy maximum relevance (mRMR) method (Peng et al., 2005) and incremental feature selection (IFS) (Liu and Setiono, 1998), was designed to select essential embedding features. These features were inputted to RAndom k-labELsets (RAKEL) (Tsoumakas and Vlahavas, 2007) using a support vector machine (SVM) (Cortes and Vapnik, 1995) or random forest (RF) (Breiman, 2001) as the base classifier to construct the multi-label classifiers. The comparison results indicated that our classifiers were superior to classifiers using traditional protein features.

## 2 Methods and Materials

This study aimed to predict the functions of mouse proteins. First, we used Word2vec and Node2vec to obtain embeddings of mouse proteins and identify the essential embedding features via the mRMR method. Then, we applied RAKEL, incorporating SVM or RF as the base classifier, to IFS to construct good multi-label classifiers.

### 2.1 Dataset

**FIGURE 1:**
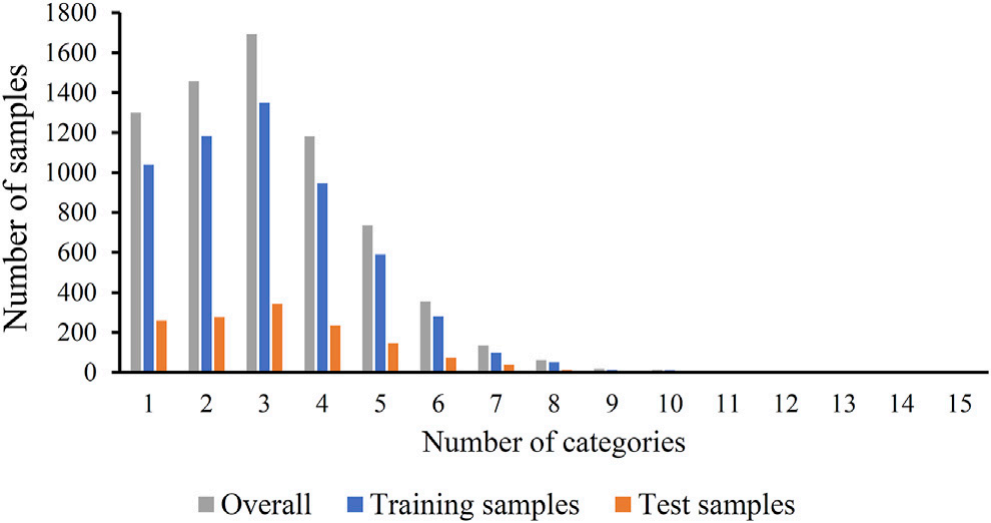
Distribution of training, test and overall samples based on the number of categories that they belong to. Several samples belong to two or more categories.

The original mouse proteins and their functions were sourced from a previous study (Hu et al., 2011), which were downloaded from MfunGD (Ruepp et al., 2006). The functions of proteins were determined by manual annotation of the literature and GO annotation (Ashburner and Lewis, 2002; Camon et al., 2003). After excluding proteins without functional domain or interaction information, 9655 proteins were obtained. These mouse proteins were further processed by CD-HIT (Fu et al., 2012) with cutoff of 0.4. 6950 mouse proteins were kept. These proteins were classified into 24 functional categories, which are listed in the second column of Table 1. In this table, the number of proteins in each category is also provided (last column of Table 1). The total number of proteins in all categories was 21584, which was higher than the number of different proteins (6950), indicating that several proteins were in more than one category. Among 6950 proteins, 1299 proteins belonged to exact one functional category, whereas others belonged to two or more categories, and no proteins belonged to more than fifteen categories. The distribution of 6950 proteins based on the number of categories that they belonged to is shown in Figure 1. Accordingly, assigning functional labels to mouse proteins was a multi-label classification problem.

**TABLE 1:** Number of proteins in each functional category.

Index	Category	Number of Proteins		
		Training dataset	Test dataset	Overall
1	METABOLISM	1152	280	1432
2	ENERGY	247	64	311
3	CELL CYCLE AND DNA PROCESSING	473	124	597
4	TRANSCRIPTION	906	229	1135
5	PROTEIN SYNTHESIS	213	45	258
6	PROTEIN FATE (folding, modification, destination)	983	234	1217
7	PROTEIN WITH BINDING FUNCTION OR COFACTOR REQUIREMENT (structural or catalytic)	3316	868	4184
8	REGULATION OF METABOLISM AND PROTEIN FUNCTION	414	102	516
9	CELLULAR TRANSPORT, TRANSPORT FACILITIES AND TRANSPORT ROUTES	915	227	1142
10	CELLULAR COMMUNICATION/SIGNAL TRANSDUCTION MECHANISM	1228	328	1556
11	CELL RESCUE, DEFENSE AND VIRULENCE	318	76	394
12	INTERACTION WITH THE ENVIRONMENT	501	138	639
13	SYSTEMIC INTERACTION WITH THE ENVIRONMENT	488	149	637
14	TRANSPOSABLE ELEMENTS, VIRAL AND PLASMID PROTEINS	3	1	4
15	CELL FATE	550	171	721
16	DEVELOPMENT (Systemic)	421	127	548
17	BIOGENESIS OF CELLULAR COMPONENTS	287	68	355
18	CELL TYPE DIFFERENTIATION	146	39	185
19	TISSUE DIFFERENTIATION	144	37	181
20	ORGAN DIFFERENTIATION	237	53	290
21	SUBCELLULAR LOCALIZATION	3920	947	4867
22	CELL TYPE LOCALIZATION	80	15	95
23	TISSUE LOCALIZATION	82	26	108
24	ORGAN LOCALIZATION	168	44	212
Sum number of proteins in all categories		17,192	4392	21,584
Number of different proteins		5560	1390	6950

To fully evaluate the final classifiers, 6950 proteins were divided into one training dataset and one test dataset, where the training dataset contained 5560 (80%) mouse proteins and the test dataset consisted of 1390 (20%) proteins. The distribution of proteins in training and test datasets based on the number of categories that they belonged to is shown in Figure 1. For convenience, the training and test datasets were denoted as *S*_*tr*_ and *S*_*te*_, respectively. The number of proteins in *S*_*tr*_ and *S*_*te*_ for each functional category is also listed in Table 1.

### 2.2 Feature Extraction

In this study, a novel feature representation scheme was presented to encode each mouse protein. This scheme extracted two types of embedding features. The first type of features was derived from functional domains of proteins, whereas the second one was obtained from a PPI network.

#### 2.2.1 Features Derived From a Functional Domain Using Word2vec

Functional domain is a type of information, which is widely used to study various protein-related problems (Cai and Chou, 2005; Xu et al., 2008; Chen et al., 2010; Zhou et al., 2017). One-hot is the classic scheme to extract features from the functional domain. In such scheme, each protein was encoded into a binary vector. However, the model based on features obtained by this scheme was quite sensitive to some domains. Here, we adopted natural language processing to extract features. The functional domain information of all mouse proteins was retrieved from the InterPro database (http://www.ebi.ac.uk/interpro/, October 2020) (Blum et al., 2021). A total of 16,797 domains were involved. Each mouse protein was annotated by at least one domain. Domains were regarded as words and proteins annotated by some domains as sentences. Word2vec (Mikolov et al., 2013; Church, 2017) was used to obtain embedding features of each domain. Its brief description was shown as follows.

Word2vec was widely used to generate word embeddings in natural language processing. It established the mapping of words to part-of-speech relationships and converted words into fixed-length real-valued vectors. The similarity of the words can be measured and characterized by the similarity of vector space. When using Word2vec, the word vector and sentence vector of features must be calculated. The probability of feature $w_{i}$ of sentence *j* in category *n* is defined as follows:

$$P_{n,j}\left(w_{i}\right)=\frac{f_{n}\left(w_{i}\right)}{\sum_{n\in N}f_{n}\left(w_{i}\right)}$$ (1)

where $f_{n}\left(w_{i}\right)$ indicates the frequency of feature $w_{i}$ in the sentence of category *n*. Then, the weight of feature $w_{i}$ can be normalized as follows:

$$\omega_{i}=\frac{\exp\left(P_{n,j}\left(w_{i}\right)+1\right)}{\sum \exp\left(P_{n,j}\left(w_{i}\right)+1\right)}$$ (2)

The sentence vector of sentence *j* in category *n* is given as follows:

$$V_{n,j}=\frac{1}{f_{j}}\sum_{i=1}^{m}\omega_{i}W\left(w_{i}\right)$$ (3)

where $f_{j}$ represents the frequency of features in sentence *j*, and $W\left(w_{i}\right)$ indicates the word vector of feature $w_{i}$ . After calculating word vector $W\left(w_{i}\right)$ and sentence vector $V_{n,j}$ of feature $w_{i}$ , the importance of feature $w_{i}$ in the sentence can be measured by the distance between the word vector and the sentence vector of feature $w_{i}$ by using the cosine distance:

$$\begin{array}{l} \text{dis}\left(V_{n,j},\;W\left(w_{i}\right)\right)=\text{cos}\left(V_{n,j},\;W\left(w_{i}\right)\right)\\ \;\;\;\;\;\;\;\;\;\;\;\;\;\;\;\;\;\;\;\;\;\;\;\;\;\;\;\;\;\;\;\;\;\;\;\;\;=\frac{V_{n,j}\;W\left(w_{i}\right)}{\left|V_{n,j}\left|*\right|W\left(w_{i}\right)\right|} \end{array}$$ (4)

The feature, whose distance value was within the scale, can be selected on the basis of the ratio of feature selection to achieve the purpose of screening and distinguishing multiple categories.

This study used the Word2vec program reported in genism (https://github.com/RaRe-Technologies/gensim). This program was performed with its default parameters. As mentioned previously, each domain was called as a word. Thus, by applying the Word2vec program, a 256-D feature vector was assigned to each domain. The feature vector of a mouse protein was defined as the average vector of feature vectors of domains, which was annotated on such protein. For convenience, such features were called domain embedding features.

#### 2.2.2 Features Derived From a Protein–Protein Interaction Network Using Node2vec

The above-mentioned embedding features of proteins were extracted from the essential properties of proteins. They cannot reflect the relationship among proteins. Recently, several network embedding algorithms, such as DeepWalk (Perozzi et al., 2014), Node2vec (Grover and Leskovec, 2016), and Mashup (Cho et al., 2016), have been proposed, which can abstract linkages in one or more networks and obtain a feature vector for each node. Features accessed in this way contained quite different information from those derived from essential properties of samples. The combination of these two types of features may fully represent each sample. To date, several models with features derived by network embedding algorithms have been set up to investigate different biological problems (Luo et al., 2017; Zhao et al., 2019; Zhou JP. et al., 2020; Pan et al., 2021a; Pan et al., 2021b; Liu et al., 2021; Zhu et al., 2021; Yang and Chen, 2022). In this study, we selected Node2vec to extract embedding features of pdsluroteins.

A network was necessary to execute Node2vec. Here, we used the PPI network reported in STRING (version 10.0) (Szklarczyk et al., 2015). The PPI information of mouse was first downloaded from STRING. Each PPI contained two proteins, encoded by Emsenbl IDs, and one confidence score. Such score was obtained by investigating several aspects of proteins, such as close neighborhood in genomes, gene fusion, occurrence across different species, gene coexpression, literature description, etc. Thus, it can widely assess the relationship among proteins. Accordingly, the PPI network used proteins as nodes, and two nodes were connected by an edge if and only if their corresponding proteins can constitute a PPI that had a confidence score larger than 0. Furthermore, we placed weight on each edge, which was defined as the confidence score of the corresponding edge. The PPI network contained 20684 nodes and 2849682 edges.

Node2vec was applied to the above-mentioned PPI network to obtain embedding features of proteins. Node2vec can be deemed as a network version of Word2vec. It produced several paths by setting each node in the network as the starting point. Each path was extended by considering the neighbors of the current end point. After generating a predefined number of paths, the nodes in each path were called as words, whereas each path was considered as a sentence. A feature vector was obtained for each node through Word2vec.

In this study, we used the Node2vec program downloaded from https://snap.stanford.edu/node2vec/. For convenience, default parameters were used. Such program was performed on the mouse PPI network. The dimension was set to 500. Finally, each mouse protein was represented by a 500-D feature vector. Features derived from PPI network via Word2vec were called network embedding features.

By combining the domain and network embedding features derived from functional domains of proteins and a PPI network, a 756-D feature vector was obtained to represent each mouse protein.

### 2.3 Feature Selection

The embedding features obtained by Word2vec and Node2vec were concatenated as the final representation of a protein. We obtained a 756-D vector for each protein. Evidently, some features may be important for assigning functional labels to mouse protein, whereas others were not. Therefore, using a feature selection procedure is necessary to screen out essential features. As several proteins had two or more functional labels, that is, they belonged to two or more functional categories, the original dataset, in which samples were assigned to multiple labels, was transformed into a new dataset in the following manner. If one sample had multiple labels, then this sample would be copied multiple times with different single labels. Then, each sample in the new dataset had only one label.

#### 2.3.1 Minimum Redundancy Maximum Relevance

All features were analyzed by the mRMR method (Peng et al., 2005). Such method evaluated the importance of features by assessing their relevance to class labels and redundancies to other features. A feature list, known as the mRMR feature list, was produced by the mRMR method. This list was produced by selecting features one by one. Initially, the list was empty. A feature with maximum relevance to class labels and minimum redundancies to features already in the list was selected and appended to the list. When all features were in the list, the procedures stopped. Evidently, features with high ranks implied that they had high relevance to class labels and low redundancies to other features. Thus, some top features in such list can comprise a compact feature space for a certain classification algorithm.

The current study used the mRMR program downloaded from http://penglab.janelia.org/proj/mRMR/. It was performed with its default parameters.

#### 2.3.2 Incremental Feature Selection

The mRMR method only generated a feature list. However, selecting the features for constructing the model remained a challenge. Here, IFS (Liu and Setiono, 1998) was used.

Given a feature list (e.g., mRMR feature list), IFS constructed all possible feature subsets. Each subset included some top features in the list. Of each feature subset, a classifier was set up and assessed by a cross-validation method (Kohavi, 1995). The feature subset with the best performance can be obtained. Features in such subset were called optimum features, whereas the classifier using these features was called the optimal classifier.

### 2.4 Multi-Label Classifier

As mentioned in Section 2.1, several proteins were in multiple functional categories. A multi-label classifier should be constructed to assign mouse proteins into functional categories. In general, two schemes were used to construct multi-label classifiers. The first one was problem transformation. It converted the original multi-label classification problem into some single-label classification problems. The second one was algorithm adaption. It extended specific single-label classifiers to deal with multi-label classification problems. This study adopted the first one to construct the multi-label classifier.

The powerful multi-label classification method, RAKEL (Tsoumakas and Vlahavas, 2007), was used to construct the multi-label classifier. Given a problem containing *l* labels (*l*=24 in this study), denoted by $L_{1},L_{2},\ldots ,L_{l}$ , RAKEL randomly produced *m* label subsets that contained *k* labels, where *m* and *k* were the main parameters of RAKEL. For each label subset, the power set was generated, and the members of this set were deemed as new labels. Based on the original labels of one sample, a new label in the power set was assigned to such sample. For example, suppose that the label subset contained three labels, say $L_{1},L_{2}$ and $L_{3}$ and a sample had three labels, say $L_{1},L_{3}$ and $L_{5}$ . In this case, this sample was assigned a new label $\left\{L_{1},L_{3}\right\}$ , which was a member of the power set of the label subset. With such operation, each sample had only one label. Accordingly, a single-label classifier with a base classifier can be set up. RAKEL integrated (*m*) such single-label classifiers as the final multi-label classifier.

This study used “RAkEL” in Meka (http://waikato.github.io/meka/) (Read et al., 2016). Such tool obtained by the RAKEL method was used to construct multi-label classifiers. The parameters *m* and *k* were all set to 10.

### 2.5 Base Classifiers

In this study, RAKEL was used to construct the multi-label classifier. It needed a base classifier to construct multiple single-label classifiers, which would be integrated into the final multi-label classifier. Here, two classic base classifiers, namely, SVM (Cortes and Vapnik, 1995) and RF (Breiman, 2001), were used, which were widely applied in tackling many biological problems (Kandaswamy et al., 2011; Nguyen et al., 2015; Chen et al., 2017; Zhou JP. et al., 2020; Zhou J.-P. et al., 2020; Liang et al., 2020; Liu et al., 2021; Onesime et al., 2021; Wang et al., 2021; Zhu et al., 2021; Chen et al., 2022; Ding et al., 2022; Li et al., 2022; Wu and Chen, 2022).

#### 2.5.1 Support Vector Machine

SVM was a supervised learning method using statistical learning theory. It can find an optimum hyperplane, which has a maximum margin between the two types of samples, in the N-dimensional space (*N* represented the number of features) using a Kernel technology (such as a Gaussian kernel), which can map data points to a given category for data classification prediction. The generalization error gradually decreased as the margin increased. A “one-to-one” strategy of SVM corresponded to the binary problem. When the problem extended to multiple classes, the strategy of SVM also changed to a “one-versus-the-rest” strategy.

This study used tool “SMO” integrated in Meka, which implemented a type of SVM. Moreover, this SVM was optimized by Sequential Minimization Optimization (SMO) algorithm (Platt, 1998). Default parameters were adopted. The kernel was a polynomial function and the regularization parameter *C* was set to 1.

#### 2.5.2 Random Forest

RF was a classic classifier used to process classification and regression problems, which was a general machine learning algorithm widely used in bioinformatics. It contained several decision tree classifiers, and subtle differences can be observed among these decision trees. RF determined its output class by aggregating votes produced by different decision trees. Compared with the decision tree, RF can avoid the overfitting problem and improve the performance.

Likewise, this study used the “RandomForest” tool integrated in Meka, which implemented RF. For convenience, default parameters were used, where the number of decision trees was set to 100.

### 2.6 Performance Measurement

K-fold cross-validation (Kohavi, 1995) is a widely used method to assess the performance of classifiers. In this method, samples are randomly and equally divided into *K* partitions. One partition is singled out as test dataset one by one, which is used to test the performance of classifier based on rest partitions. Accordingly, each sample is tested only once. The comparison of predicted labels and true labels can lead to some measurements to indicate the performance of classifiers. In this study, we selected 10-fold cross-validation to test all multi-label classifiers.

After the 10-fold cross-validation, each sample was assigned with one or more labels. Some measurements can be computed to assess the predicted results. As a multi-label classifier, accuracy and exact match were the widely used measurements (Zhou JP. et al., 2020; Zhou J.-P. et al., 2020; Pan et al., 2021b; Chen et al., 2021; Tang and Chen, 2022). They can be calculated using the following equations:

$$\left\{ \begin{array}{l} \text{Accuracy}=\frac{1}{n}\sum_{i=1}^{n}\left(\frac{\text{||}L_{i}\cap L_{i}^{\prime }\text{||}}{\text{||}L_{i}\cup L_{i}^{\prime }\text{||}}\right)\\ \text{Exact match}=\frac{1}{n}\sum_{i=1}^{n}\Theta \left(L_{i}\text{,}L_{i}^{\prime }\right) \end{array}\right.$$ (5)

where *n* stands for the number of samples; $L_{i}$ and $L_{i}^{'}$ denote the set consisting of true labels and predicted labels of the *i*-th sample, respectively; $\Theta \left(L_{i},L_{i}^{'}\right)$ is defined as follows:

$$\Theta \left(L_{i},L_{i}^{\prime }\right)=\{\begin{array}{cc} 1, & \text{If}\;L_{i}^{\prime }\;\text{is}\;\text{identical}\;\text{to}\;L_{i}\\ 0, & \text{Otherwise}\text{.} \end{array}$$ (6)

Evidently, the higher the accuracy or exact match, the higher the performance.

## 3 Results and Discussion

**FIGURE 2:**
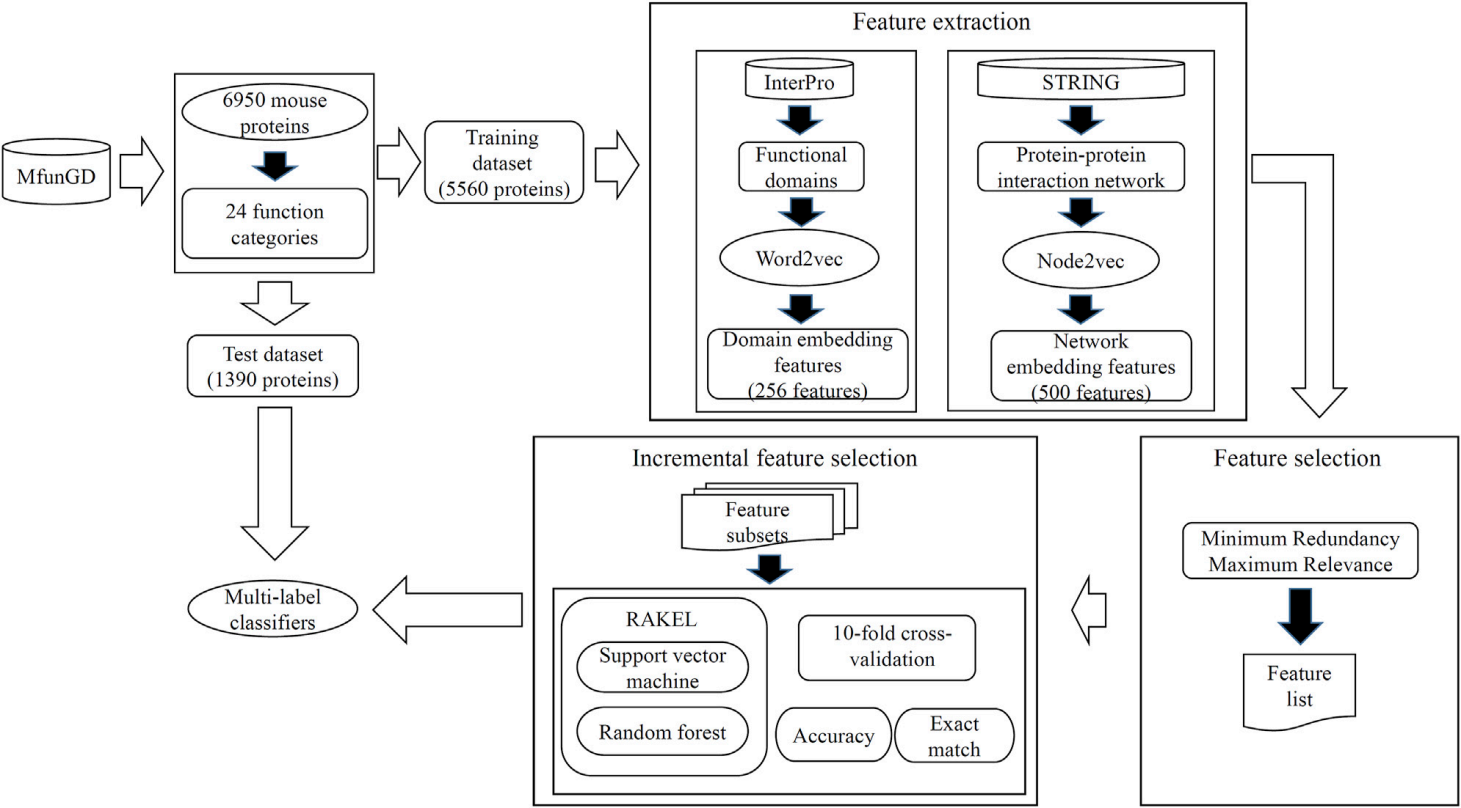
Entire procedures to construct the multi-label classifiers for predicting functions of mouse proteins. Mouse proteins and their function annotations are retrieved from MfunGD. These proteins are randomly divided into one training dataset and one test dataset. Embedding features were derived from protein functional domains and protein–protein interaction network through Word2vec and Node2vec, respectively. A feature selection procedure is used to analyze embedding features, and essential features are fed into RAKEL to construct the multi-label classifiers. Proteins in the test dataset are fed into these classifiers to further evaluate their performance.

In this study, some novel multi-label classifiers were proposed to identify the functions of mouse proteins. The entire procedures are shown in Figure 2. In this section, we provided the detailed results of all procedures and made some comparisons to elaborate the unity of the classifier.

### 3.1 Results of the mRMR Method on Training Dataset

Each protein in *S*_*tr*_ was represented by 756 embedding features. These features were analyzed by the mRMR method, resulting in a feature list, which is called the mRMR feature list. This list is provided in Supplementary Table S1.

### 3.2 Results of IFS on Training Dataset

**FIGURE 3:**
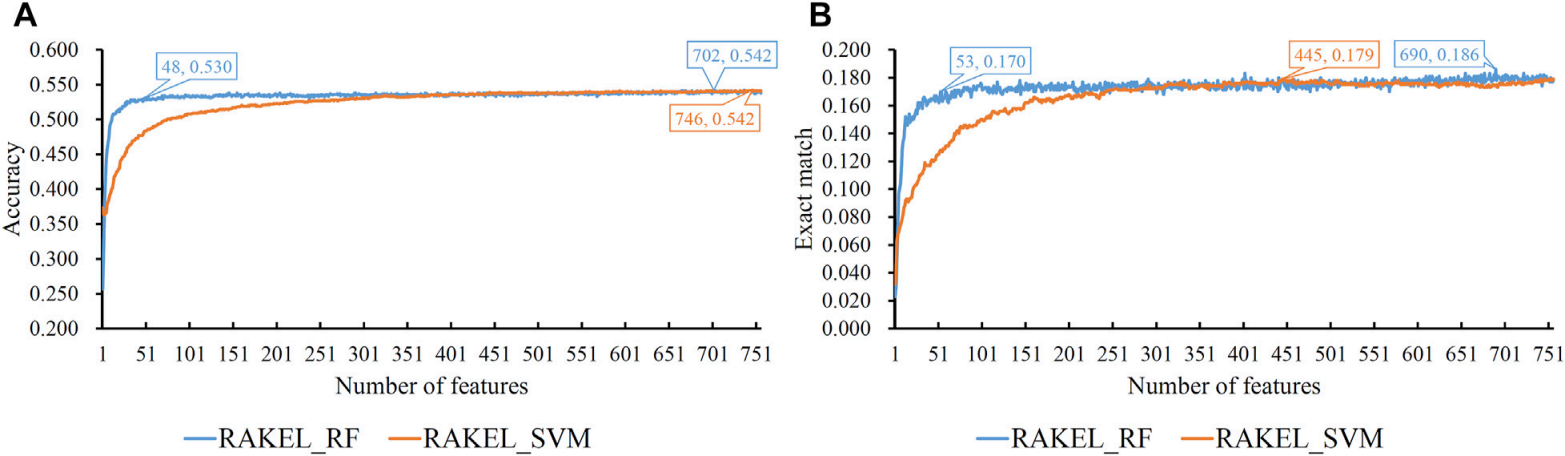
IFS curves on embedding features using different classification methods. **(A)** Accuracy is set to the *Y*-axis. **(B)** Exact match is set to the *Y*-axis. RAKEL_RF/RAKEL_SVM indicates that RAKEL with RF/SVM as the base classifier is used to construct the multi-label classifiers.

Based on the mRMR feature list provided in Supplementary Table S1, IFS was used to construct several feature subsets and set up a multi-label classifier on each feature subset. Each multi-label classifier was set up with RAKEL, and the SVM or RF was selected as the base classifier. 10-fold cross-validation was used to assess the performance of each classifier. The predicted results were assessed by calculating the accuracy and exact match, as mentioned in Section 2.6, which are available in Supplementary Table S2. Some IFS curves are plotted in Figure 3 to show the performance of multi-label classifiers with different base classifiers and feature subsets, where the *X*-axis represented the number of features, and the *Y*-axis represented the accuracy or exact match.

As shown in Figure 3A, when the base classifier was RF, the highest accuracy was 0.542, which was produced by using the top 702 features in the list. Thus, we can construct an optimum multi-label classifier with these features and RF. As for another base classifier SVM, the highest accuracy was also 0.542, which was produced by using the top 746 features. An optimum multi-label classifier with SVM can be built using these features. Above two optimum classifiers provided the same accuracy. However, the exact match of the classifier with RF was 0.182 and that of the classifier with SVM was 0.179. Accordingly, the optimum multi-label classifier with RF can be deemed to be superior to the optimum multi-label classifier with SVM. When accuracy was used as the key measurement, we can construct a multi-label classifier using the top 702 features and RF. However, the efficiency of such classifier was not very high because lots of features were involved in such classifier. From Figure 3A, we can see that the IFS curve of RF followed a sharp increasing trend when a few features were used. It can quickly provide a quite high accuracy using much less features than SVM. By carefully checking accuracy listed in Supplementary Table S2 and Figure 3A, we can find that when top 48 features were adopted, the classifier with RF can yield the accuracy of 0.530, which was only a little lower than that of the optimum classifier. Such classifier can be picked up as a tool to predict functions of query mouse proteins.

For the exact match, two IFS curves corresponding to two different base classifiers are plotted in Figure 3B, from which we can see that the base classifier RF generated the highest exact match of 0.186 when the top 690 features were used, whereas SVM yielded the highest exact match of 0.179 when the top 445 features were used. Evidently, the best multi-label classifier with RF was superior to the best multi-label classifier with SVM when exact match was regarded as the key measurement. Accordingly, we can construct a multi-label classifier using the top 690 features and RF. The same problem also existed for such classifier, i.e., low efficiency. It can be observed from Figure 3B that the IFS curve of RF was quite similar to that in Figure 3A **.** The increasing trend was much sharper at the beginning of the curve than that of IFS curve of SVM. This meant that RF can provide a high exact match using a small number of features. When top 53 features were used, the classifier with RF can produce exact match of 0.170, which was a little lower than that of the best multi-label classifier with RF. Accordingly, such classifier can be an efficient tool to identify functions of mouse proteins.

As previously mentioned, different key measurements can lead to different multi-label classifiers. For different prediction purposes, users can select the key measurement and use the corresponding classifier. The performance of above-mentioned classifiers is listed in Tables 2, 3.

**TABLE 2:** Accuracy of the important multi-label classifiers with different features on training and test datasets.

Method	Feature	Number of Features	Accuracy	
			Training dataset	Test dataset
RAKEL_RF	Embedding features	702	0.542	0.536
RAKEL_SVM	Embedding features	746	0.542	0.537
RAKEL_RF	Embedding features	48	0.530	0.530
RAKEL_RF	Domain features	26	0.429	0.426
RAKEL_SVM	Domain features	27	0.429	0.428
RAKEL_RF	Linkage features	233	0.462	0.460
RAKEL_SVM	Linkage features	234	0.432	0.424
RAKEL_RF	Domain and linkage features	221	0.470	0.462
RAKEL_SVM	Domain and linkage features	227	0.449	0.433

**TABLE 3:** Exact match of the important multi-label classifiers with different features on training and test datasets.

Method	Feature	Number of Features	Exact match	
			Training dataset	Test dataset
RAKEL_RF	Embedding features	690	0.186	0.171
RAKEL_SVM	Embedding features	445	0.179	0.157
RAKEL_RF	Embedding features	53	0.170	0.159
RAKEL_RF	Domain features	25	0.077	0.078
RAKEL_SVM	Domain features	29	0.075	0.077
RAKEL_RF	Linkage features	158	0.130	0.123
RAKEL_SVM	Linkage features	225	0.113	0.104
RAKEL_RF	Domain and linkage features	201	0.135	0.130
RAKEL_SVM	Domain and linkage features	215	0.132	0.111

### 3.3 Distribution of Embedding Features Used in Two Efficient Classifiers

**FIGURE 4:**
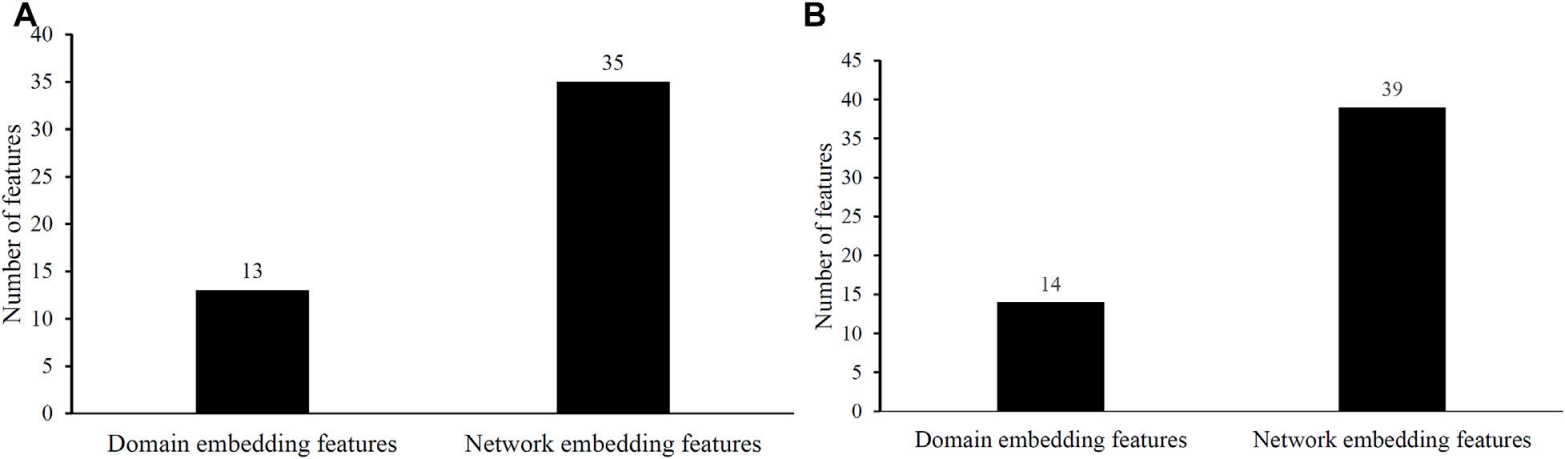
Distribution of embedding features used in two efficient classifiers. **(A)** Distribution of embedding features used in the classifier selected by accuracy. **(B)** Distribution of embedding features used in the classifier selected by exact match.

Two efficient classifiers were constructed as mentioned above, which can be efficient tools for identification of protein functions. 48 and 53 embedding features were involved in these two classifiers, respectively. Their distributions on domain and network embedding features are shown in Figure 4. For the classifier with 48 features, 13 were domain embedding features, whereas 35 were network embedding features. As for that with 53 features, similar results can be observed (14 for domain embedding features and 39 for network embedding features). These results indicated that network embedding features gave more contributions for constructing two classifiers. However, domain embedding features were also important. Their combination was one important reason why these two classifiers yielded such good performance.

### 3.4 Performance of Classifiers on Test Dataset

Based on accuracy and exact match, three multi-label classifiers were built, respectively. These classifiers were further evaluated on *S*_*te*_. Their performance is listed in Tables 2, 3. For the three classifiers selected by accuracy, the optimum classifiers with RF or SVM yielded the accuracies of 0.536 and 0.537 (Table 2), respectively, which were slightly lower than those on *S*_*tr*_. The accuracy of the efficient classifier with RF produced the accuracy of 0.530 (Table 2), same as that on *S*_*tr*_. These results indicated that the generalization of these classifiers was quite good. As for the three classifiers selected by exact match, they provided exact match values of 0.171, 0.157 and 0.159 (Table 3), respectively. They were lower than those on *S*_*tr*_. However, the decrease was in an acceptable range. Thus, the generalization of these classifiers was also satisfied.

### 3.5 Comparison With Other Classifiers

In this study, we adopted a novel set of features to represent each mouse protein and constructed some multi-label classifiers to predict their functions. This section adopted some classic features to construct the classifiers and make some comparisons.

Two types of embedding features were used in this study. They were derived from the protein functional domain and PPI network. For the protein functional domain, the classic usage of encoding proteins was the one-hot scheme. In detail, a protein was encoded into a binary vector under such scheme. Each domain was used as a dimension, and the component was set to one if the protein had the corresponding domain annotation; otherwise, the component was set to zero. Here, 16797 domains were involved, inducing a 16797-D binary vector for each mouse protein. For an easy description, these features were called as domain features in this study. As for the PPI network, such information can be directly used by selecting all linkages between a protein and all proteins in the network and collecting them in a vector to encode the protein. Accordingly, each mouse protein was represented by a 20684-D vector, as 20684 proteins were found in the PPI network. These features were called as linkage features. Each mouse protein was represented by domain features or linkage features or both of them, inducing three representations of proteins. We investigated the performance of classifiers on each protein representation.

**FIGURE 5:**
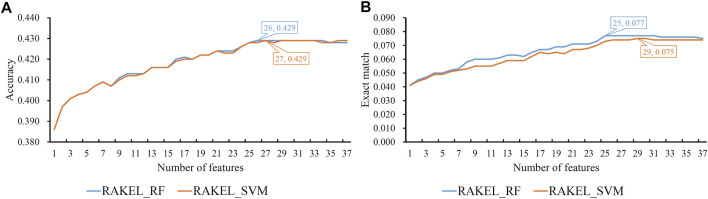
IFS curves on domain features using different classification methods. **(A)** Accuracy is set to the *Y*-axis. **(B)** Exact match is set to the *Y*-axis. RAKEL_RF/RAKEL_SVM indicates that RAKEL with RF/SVM as the base classifier is used to construct the multi-label classifiers.

**FIGURE 6:**
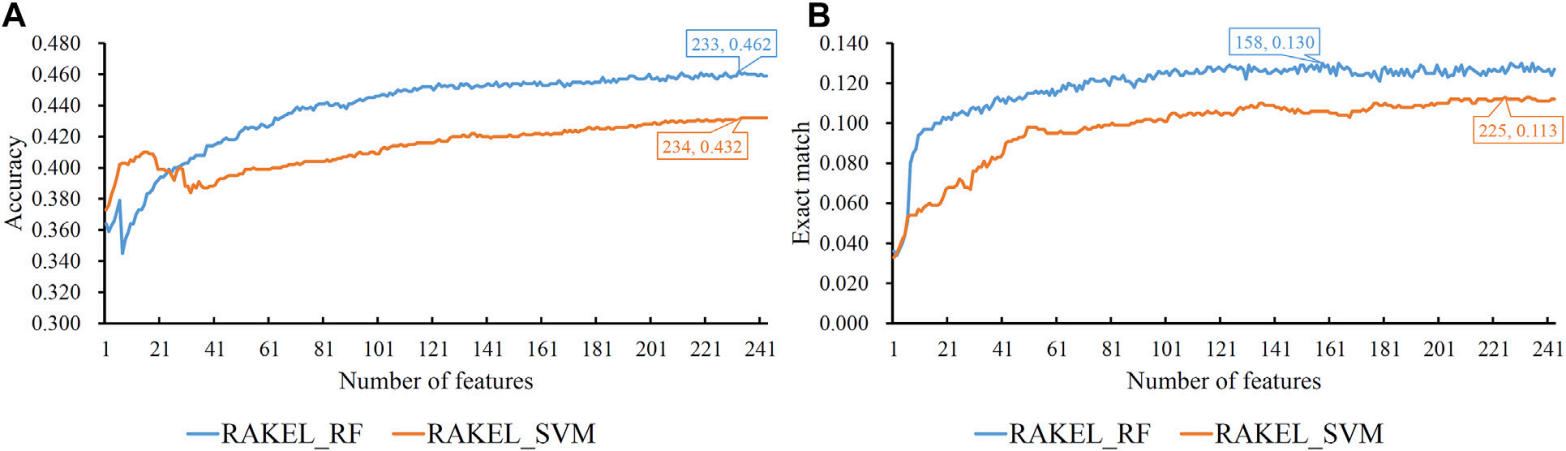
IFS curves on linkage features using different classification methods. **(A)** Accuracy is set to the *Y*-axis **(B)** Exact match is set to the *Y*-axis. RAKEL_RF/RAKEL_SVM indicates that RAKEL with RF/SVM as the base classifier is used to construct the multi-label classifiers.

**FIGURE 7:**
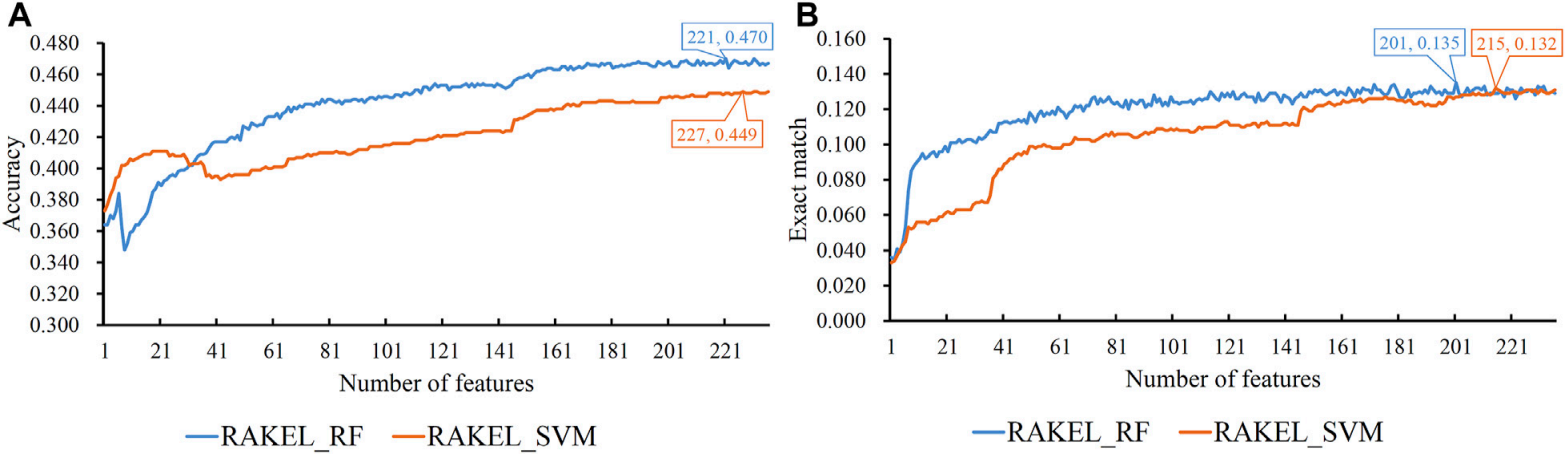
IFS curves on domain and linkage features using different classification methods. **(A)** Accuracy is set to the *Y*-axis. **(B)** Exact match is set to the *Y*-axis. RAKEL_RF/RAKEL_SVM indicates that RAKEL with RF/SVM as the base classifier is used to construct the multi-label classifiers.

As previously mentioned, proteins were represented by lots of features in each representation. A feature selection procedure was necessary. However, given the large number of features, we first adopted Bortua (Kursa and Rudnicki, 2010; Zhang et al., 2021) to exclude irrelevant features. 37 and 243 features were selected by Bortua for domain and linkage feature representations, respectively. When domain and linkage features were combined together to encode mouse proteins, 236 features were kept by Bortua. Then, these remaining features were evaluated by the mRMR method, resulting in an mRMR feature list for each representation. IFS was used to construct optimum multi-label classifiers for accuracy and exact match. We still used RAKEL to construct the classifiers, and SVM or RF was selected as the base classifier. The IFS results are provided in Supplementary Tables S3-S5. Likewise, some IFS curves are plotted in Figures 5–7.

The best accuracies for different base classifiers on *S*_*tr*_ are listed in Table 2, in which those obtained by embedding features are also provided. When the base classifier was RF, the accuracies obtained by domain features, linkage features and both of them were all lower than 0.5, which were much lower than those of the classifiers on embedding features. Furthermore, the base classifier (SVM) yielded similar results (see Table 2). As for the exact match, the best values for different base classifiers are listed in Table 3, in which those obtained by embedding features are also listed. Evidently, the exact match obtained by embedding features was also higher than that obtained by domain features or linkage features or both of them regardless of the base classifier used (RF or SVM). The improvement was at least 3%. Furthermore, from Tables 2, 3, the classifiers with embedding features also yielded better performance on test dataset *S*_*te*_ than those with domain features or linkage features or both of them. All above results indicated that the novel features used in this study were more efficient than the features produced by traditional methods in predicting protein functions. In addition, it can be observed from Tables 2, 3 that when domain and linkage features were combined to represent proteins, the classifiers were always better than those only using domain features or linkage features. This fact indicated that combination of the domain and network information of proteins can improve the performance of classifiers. These two types of information can complement each other in predicting functions of proteins.

## 4 Conclusion

In this paper, we proposed some multi-label classifiers to predict the functions of mouse proteins. These classifiers adopted novel features, which were derived from protein functional domains and the PPI network via word embedding and network embedding, respectively. The performance of the classifiers was better than those using features extracted by traditional methods, thereby indicating that the novel features have stronger discriminative power. Therefore, the newly proposed classifiers can be used to predict protein functions, and such novel features can be used to tackle other protein-related problems.

## Data Availability

Publicly available datasets were analyzed in this study. This data can be found here: http://mips.gsf.de/genre/proj/mfungd.

## Supplementary Material

Supplementary Table S1 mRMR feature list yielded by the mRMR method on training dataset.

Supplementary Table S2 IFS results with embedding features on training dataset.

Supplementary Table S3 IFS results with domain features on training dataset.

Supplementary Table S4 IFS results with linkage features on training dataset.

Supplementary Table S5 IFS results with domain and linkage features on training dataset.
